# Does green credit policy affect corporate innovation performance?—A quasi-natural experiment based on Green Credit Guidelines

**DOI:** 10.1371/journal.pone.0291764

**Published:** 2023-10-04

**Authors:** Jingjing Wu, Qingxing Tang, Yi Yang

**Affiliations:** 1 College of Foreign Studies, Guilin University of Technology, Guilin, Guangxi, China; 2 Institute for Regional Economic and Language Service Research, Guilin University of Technology, Guilin, Guangxi, China; 3 School of Economics and Management, Guangxi University of Science and Technology, Liuzhou, Guangxi, China; Government College University Faisalabad, PAKISTAN

## Abstract

For urgent need to the transition to sustainable development, it is of great significance to explore the driving role of green credit policies in innovation performance. This study uses a sample of Chinese A-share listed companies from 2004 to 2019 and constructs a quasi-natural experiment based on the Green Credit Guidelines issued by the China Banking Regulatory Commission in 2012. PSM-DID method is employed to examine the innovation impact of green credit policies in both green credit-restricted and non-restricted industries, thereby exploring the its mechanism of influence on firm innovation performance. The results show that after the implementation of Guidelines, it promotes a company’s innovation output, significantly enhances its innovation performance, especially in terms of quantity-based incentives. However, the incentive effect on quality-based incentives is relatively limited. Secondly, by increasing the level of risk-taking within the enterprise and strengthening external environmental regulations, the green credit policy can further enhance its promotion effect on corporate innovation performance. Finally, the promotion effect of green credit policies is more significant for state-owned enterprises and large-scale enterprises. The research results help to break through the bottleneck of corporate innovation with the coordination of environmental protection and economy, which further improve the sustainability of economic development.

## 1. Introduction

With the deepening development of industrialization and urbanization, environmental pollution has been increasing [1]. It is urgent to break away from the traditional production mode with high energy consumption and emission, wich overly relies on resource consumption. The proposals of carbon peak and carbon neutrality [2] also officially declare that the unsustainable economic development model relies on resource consumption in China, which comes to a time for transformation. In the shift from heavy speed to quality-oriented development, China has already invested a lot of resources in taxes and administrative measures [3], but it still has not achieved the ideal goals in environmental optimization. Since the current governance measures in China include pollution discharge fees, desulfurization and denitrification, which cannot directly constrain the total carbon emissions [4, 5]. To achieve fundamental environmental protection, it is essential to combine financial instruments with end-of-pipe treatment methods effectively [6]. In 2012, the China Banking Regulatory Commission (CBRC, renamed as the Financial Regulatory Bureau) officially issued the Green Credit Guidelines (hereafter Guidelines), which provided a good perspective for measuring the development of green finance. Guidelines standardize the green credit of financial institutions such as banks, and raised the requirements for corporate environmental management. Thus, the innovation output of enterprises has increased in various regions, which is reflected in the growth of national patent numbers in environmental management. Guidelines incorporate the environmental performance of enterprises into the loan assessment standards of banks, and then re-adjuste resource allocation. Innovation can fundamentally reduce pollutant production by improving process technology and optimizing production structure, etc., thereby achieving a higher efficiency in pollution control and environmental optimization.

In oder to achieve the dual goals of stable growth and environmental optimization, China economy can transform towards green environmental protection by green finance to guide resources [7]. By introducing limited financial resources into specific environmental protection fields, environmental governance investment will inevitably have a crowding-out effect on productive investment in the short term [8]. However, in the long run, the benefits brought by innovation can effectively offset the environmental protection investment, and achieve an increase in capital efficiency through the compensation effect [9]. Secondly, the increasingly severe financing constraints make enterprises achieve emission reduction targets only through technological transformation. Finally, due to issues such as insufficient market-based tools or behavioral incentives for micro-subjects, they will greatly increase the operational difficulties. However, the advantages of finance in funds can effectively alleviate such problems [10]. Therefore, green finance is an important driver for the dual carbon target, and the main direction of future financial institution [11]. Although a series of policies on green finance and corporate innovation have been introduced before, there is still a lack of research on how to effectively combine the two, and the underlying mechanism remains to be further explored.

This paper takes green credit as a representative of green finance to explore the impact and mechanism of green credit policies on corporate innovation performance. Taking the green credit policy as an exogenous shock variable, the PSM-DID model is used to investigate its different effects on innovation performance between green credit-restricted and non-restricted industries, and empirically analyze the motivation and restraint of Guidelines on corporate innovation performance. Secondly, this paper further explores the potential regulatory mechanisms, which is also the innovation point of this study. Many complex uncertainties, as the long cycle, large investment and uncertain conversion of innovation, may have important influences on innovation decision-making. Enterprises have dual roles as economic part and social part. As the external pressure, the government environmental regulation may stimulate or force companies to change the traditional production methods with high energy consumption and emission, thus achieving technological transformation and upgrading. From the risk-taking level of enterprise and environmental regulation, the paper studies the potential mechanisms that affect the actual effectiveness of green credit policies, in order to explore better ways to play the role of green finance in supporting corporate innovation performance.

There are some potential marginal contributions of this study: 1) deepening the integration of green credit and innovation-driven development, the study explores the actual effects and problems of green credit policies on corporate innovation performance, and provides references for further improvement of relevant theories and system construction; 2) Most present studies have explored the role of financing constraints, but innovation uncertainty and other regulatory tools are lack of consideration. Matching government environmental regulation with the risk-taking and innovation capabilities of enterprise, the study finds out the innovation constraints faced by enterprises, and provides diversified paths for the green credit policies in coordinating environmental protection and economic goals.

## 2. Research hypothesis

### 2.1 Green credit policy and innovation

China has been formally implementing the green credit policy since 2007, and the issuance of Guidelines transformed it from a voluntary to a mandatory policy in 2012, establishing a series of standards for financial institutions and enterprises. Green credit differs from other types of policies as it introduces resources into the clean industry field to achieve environmental governance goals. In the process of green credit, financial institutions incorporate enterprise environmental performance into the credit assessment system, and use loan interest rates to control enterprise funding sources, thereby influencing innovative behaviors such as R&D investment. Moreover, companies with good environmental risk management often receive higher return quotas, which attract more investors to invest in enterprises with better environmental performance.

The impact of green credit policy is mainly achieved through its incentive and constraint effects. Firstly, it incentivizes clean industries by incorporating enterprise environmental risk management into the credit assessment system, which controls the financing opportunities for enterprises [12]. As a result, enterprises with better performance are more likely to obtain funding opportunities at a lower cost of capital, thereby gaining more social resources. Secondly, with increasingly stringent external environmental pressures [13] in the long run, enterprises face significant disparities between high costs and low effects. This makes them more likely to achieve environmental goals and high profit margins through innovative production technologies [14]. Thirdly, it constrains polluting industries, imposing higher financing thresholds and costs during the credit process for enterprises with poor performance or those that have caused extreme environmental events. In order to obtain sustainable and stable financing sources, polluting enterprises are forced to transform and upgrade their operations with technological innovation [15]), in order to reduce the risks and costs of environmental management [16]. In conclusion, from the perspective of resources and costs, green credit policy can guide enterprises to achieve innovation transformation by controlling resource flow and pressure output, whether it is due to resource effects or cost effects [17, 18]. On the one hand, enterprises want to obtain more resource allocation with better “green” performance. On the other hand, they prefer to reduce the cost of environmental governance and get funding support at a lower financing cost, thereby the process promotes the innovation performance. Based on these observations, the paper propose the following hypothesis:

Hypothesis 1: The implementation of Green Credit Guidelines has a significant positive impact on innovation performance in green credit-restricted industries.

### 2.2 Moderation effect analysis

#### 2.2.1 Analysis in the moderating mechanism of risk-taking level in enterprise

Easing financing constraints, Guidelines provide some enterprises with opportunities to obtain more borrowing funds, and promotes their innovation investment. However, subjective foresight is the main factor guiding behavior in reality, but the future is full of uncertainties [19]. Therefore, risk-taking is indispensable during the enterprise innovation [20]. Due to a lack of knowledge and experience, decision-makers may rely solely on past data to evaluate innovative projects, making the results uncertain [21]. On one hand, the market is constantly changing; on the other hand, the maturity of technology and public acceptance are still to be tested. These two factors lead to uncertainty about future predictions, resulting in risks of ineffective innovation activities [22]. Therefore, when the risk-taking level is low, enterprises often reduce challenging projects to avoid unnecessary or excess risks. They are more inclined to choose low-risk and low-return investment projects to maintain the status quo rather than challenge innovation [23]. If the risk-taking level of an enterprise is high, the more mature risk prevention mechanisms can provide protection and compensation for risky projects, giving enterprise managers more confidence in innovative projects [24]. This also makes the enterprise easier to attract investment and social resources. Based on signaling theory, the higher the enterprise’s interest in innovative projects, the greater the investment in innovative research and development. Based on this, the paper proposes the following hypothesis:

Hypothesis 2: The higher the risk-taking level is, the more significant the promotion of innovation is after the implementation of Green Credit Guidelines.

#### 2.2.2 Analysis in the moderating mechanism of environmental regulations

Environmental regulation is a crucial governmental tool for the environment protection, playing a significant role in reducing CO_2_ emissions and the probability of extreme events [25]. While some argue that environmental regulations increase enterprises’ cost burden, but appropriate regulations can drive them to promote green transformation with new production technologies. The Porter hypothesis [26] suggests that suitable environmental regulations can help enterprises achieve greater profitability, and generates an innovation compensation effect for increased environmental governance costs [27]. Since environmental regulations can improve waste reuse rates, and indirectly promote enterprise technological innovation by R&D investment and human capital. When environmental policies exert significant pressure on enterprises, they tend to eliminate outdated capacity and improve products or technologies with innovative methods. When the environmental regulation is weak, enterprises are more likely to direct pollution, which significantly reduce their treatment costs [28]. Conversely, when the environmental regulation is strong, the innovation compensation effect can offset internal governance costs, making the benefits from technological innovation higher than the input costs [29, 30]. Based on Porter’s hypothesis of innovation compensation effect, the increase in the compliance costs may lower capital efficiency in the short term, but long-term accumulation may compensate for the increased costs. In this way, enterprises achieve the economic goals with the increasing external environmental supervision, which stimulates their innovation willingness. Based on this analysis, the paper proposes the following hypothesis:

Hypothesis 3: The stronger the environmental regulation is, the more significant the innovation effect will be after the implementation of Green Credit Guidelines.

### 2.3 Heterogeneity analysis

#### 2.3.1 Heterogeneity analysis based on enterprise property rights

Due to China’s unique institutional background, there may be some differences between state-owned enterprises and non-state-owned enterprises in terms of resource acquisition, which may result in uneven innovation output. Therefore, this paper further analyzes the impact of green credit policies on innovation output from the perspective of enterprise property rights. State-owned enterprises are more likely to obtain the trust of financial institutions such as banks due to government support, as well as funding at lower thresholds and costs [31]. However, for non-state-owned enterprises, the green credit policies still cannot alleviate the inherent impression that banks have on private enterprises [32], which often results in stricter financing conditions and greater financing difficulties [33]. In addition, state-owned enterprises are more agile in government policy than private enterprises, and thus the policy effect in state-owned enterprises may be better [34]. Based on this analysis, the paper proposes the following hypothesis:

Hypothesis 4: The promotion effect on innovation performance will be more significant for state-owned enterprises after the implementation of Green Credit Guidelines.

#### 2.3.2 Heterogeneity analysis based on enterprise size

According to the Schumpeterian hypothesis, enterprise size are also factors that affect whether companies undergo technological change [35]. Because of the information asymmetry, enterprise size is a kind of decision-making information, which is more easily obtained for external resource providers such as banks than the internal-oriented information such as R&D [36]. Compared with small-scale enterprises, large-scale enterprises typically have more mature technology development capabilities, internal control measures and richer resource reserves, wihch provide a more stable market position, greater recognition and resistance of risks. Such advantages enable large-scale enterprises to weaken the dilution effect of technical spillovers on the exclusivity of innovation benefits [37]. Within the start-up period or lacking sufficient resources such as funds, small-scale enterprises usually result in insufficient internal and external incentives, which may lead to lower innovation enthusiasm [38], and thus decision-makers will try to avoid potential losses from technological innovation, whereas large-scale enterprises are more confident and capable of technological innovation. Based on this analysis, the paper proposes the following hypothesis:

Hypothesis 5: The promotion effect on innovation performance will be more significant for large-scale enterprises after the implementation of Green Credit Guidelines.

## 3. Research design

### 3.1 Sample selection and data sources

This paper examines all A-share listed companies from 2004 to 2019, using data sources such as the Guotai-An (CSMAR) database and China Environmental Statistics Yearbook. To ensure accuracy, the study excludes certain samples based on the following criteria: (1) exclude listed companies in the financial industry; (2) exclude ST, *ST and companies with missing important variables; (3) truncate continuous variables in the model at the 1% and 99% levels to exclude the influence of outliers. Data processing is completed with Stata16.0.

### 3.2 Model construction and indicator selection

Compared with static comparison or traditional difference methods, the difference-in-difference method (DID) compares the difference before and after the unimplemented group with the difference before and after the implemented group, which can effectively avoid endogeneity issues of policy as an explanatory variable. The paper takes panel data of A-share listed companies as the research sample and controls for unobservable individual and time heterogeneous factors that may affect innovation performance by leveraging the exogeneity of the explanatory variable.

The DID method was used to investigate the effect of green credit on innovation, as shown in the following equation:

$$Patent_{it}=\alpha_{t}+\beta_{1}Post_{t}+\beta_{2}Treat_{i}\times Po{\mathrm{st}}_{t}+\beta_{3}Treat_{i}+\gamma^{'}x_{it-1}+\varepsilon_{i,t}$$ (1)

#### a. Dependent variable: Innovation performance (Patent)

The dependent variable Patent_it_ represents the innovation performance of the i-th listed company in the t-th year measured by the number of patent applications. To measure the quality and quantity of enterprise innovation, the paper uses the number of invention patent applications (Inva), utility model patent applications (Uma), and the total number of patents (Total). To address the problem of right-skewed distribution of the dependent variable and eliminate the impact of extreme data, the paper adds 1 to the number of patent applications and takes the logarithm to obtain LnTotal, LnInva, and LnUma.

#### b. Core explanatory variable: Post×Treat

The core explanatory variable is the green credit policy, industry attributes, and their interaction term. To satisfy the requirements of the double difference method, two dummy variables, Post and Treat, were set. Post is a time variable; it is set to 0 before the implementation of Green Credit Guidelines (t<2012) and set to 1 after its implementation (t≥2012). Treat indicates whether the i-th company belongs to the green credit-restricted industry. Industries identified as A-class risks in the Green Credit Guidelines are considered to be restricted by green credit policies. The experimental group (Treat = 1) consists of companies in these industries, while the control group (Treat = 0) consists of companies not in these industries.

#### c. Moderator variables: Risk-taking level and environmental regulation

Risk-taking level of enterprises: the paper uses earnings volatility as the most commonly used measure of enterprise risk-taking. The higher the degree of earnings volatility, the higher the level of risk-taking, and vice versa. Drawing on existing research, Roa is adjusted by annual-industry mean to mitigate the effects of economic cycles and industry factors.


$$Adj\_Roa_{\mathrm{it}}=\frac{EBITDA_{it}}{ASSETS_{it}}-\frac{1}{X}\sum_{k=1}^{X}\frac{EBITDA_{kt}}{ASSETS_{kt}}$$ (2)

$$R{\mathrm{isk}}_{\mathrm{it}}=\sqrt{\frac{1}{T-1}\sum_{t=1}^{T}{\left(A\mathrm{dj}\_Roa_{it}-\frac{1}{T}\sum_{t=1}^{T}A\mathrm{dj}\_Roa_{it}\right)}^{2}}/T$$ (3)

Environmental regulation: Environmental protection investment (EPI) is a direct government investment in environmental management in various regions, which is the main means of governance in China. Thus, the paper uses EPI/GDP as the measurement indicator to avoid measurement errors caused by regional economic differences. The larger the investment of environmental protection is, the greater the environmental regulation in the region where the enterprise is located.

#### d. Control variables

Drawing on existing research, the paper includes a series of control variables X_i, t-1._ to control for other individual characteristics that may affect innovation performance of companies. These variables include enterprise size (Size), Return on Assets (Roa), debt level (Lev), cash ratio (Cash), years since establishment (Age), proportion of independent directors (Ind), CEO duality (Dual), Institutional Ownership (Inst), number of employees (Employee), revenue growth rate (Growth), free cash flow (Fcf), separation of ownership and control (Sep), book-to-market ratio (MtB), number of board members (Board), and fixed asset ratio (Ppe) in Table 1.

**Table 1:** Variable description.

Variable Category	Variable Name	Variable Symbol	Variable Definition
Dependent Variable	Patent applications	Total	Firm’s total number of patent applications
	Invention patents	Inva	Firm’s number of invention patent applications
	Practical new-type patents	Uma	Firm’s number of practical new-type patent applications
Explanatory Variables	Green credit policy	Post	A time variable that equals 1 for years after 2012 and 0 otherwise
	Restricted industries by green credit	Treat	A dummy variable that equals 1 if the firm is restricted by the green credit policy and 0 otherwise
Control Variables	Firm Size	Size	Natural logarithm of year-end total assets
	Return on Assets	Roa	Ratio of net profit to total assets at the end of the year
	Debt level	Lev	Total debt divided by total assets
	Cash ratio	Cash	End-of-year monetary funds divided by year-end total assets
	Years since establishment	Age	Difference between the current year and the year of the firm’s IPO
	Proportion of independent directors	Ind	Number of independent directors divided by the total number of board members
	CEO duality Dual	Dual	A dummy variable that equals 1 if the CEO also serves as chairman of the board, and 0 otherwise
	Institutional Ownership	Inst	Ratio of institutional shares to total shares outstanding
	Employment size	Employee	Number of employees
	Revenue growth rate	Growth	Annual increase in operating revenue
	Free cash flow	Fcf	Net cash flow from operating activities divided by total assets
	Separation of ownership and control	Sep	Difference between the controlling rights and ownership rights
	Book-to-market ratio	MtB	Equity value divided by market capitalization
	Number of board members	Board	Total number of board members
	Fixed asset ratio	Ppe	Ratio of fixed assets/total assets

## 4. Empirical analysis

### 4.1 Descriptive statistics

Table 2 presents the descriptive statistics for the variables. The mean value of the policy dummy variable Post is 0.678, indicating that nearly 70% of the sample companies applied for patents after the implementation of the green credit policy. The Treat dummy variable, which represents industries restricted by the Guidelines, has a mean value of 0.189, suggesting that the innovation performance of 18.9% of the companies was affected to some extent after the introduction of the green credit policy. The dependent variable, total patent applications, has an average value of 2.026, with a standard deviation of 1.743, indicating significant variation among the innovative performance of different listed companies. Moreover, the mean values of LnInva and LnUma are 1.418 and 1.543, respectively, suggesting that enterprises produce more practical patents. Moreover, both variables have a standard deviation of approximately 1.5, highlighting the significant differences in both the quantity and quality of practical and invention patent applications among different companies.

**Table 2:** Descriptive statistics of main variables.

	N	Mean	Std.Dev.	Min	Max
LnTotal	30,915	2.026	1.743	0.000	6.506
LnInva	30,915	1.418	1.465	0.000	5.717
LnUma	30,915	1.543	1.602	0.000	5.932
Post	30,915	0.679	0.467	0.000	1.000
Treat	30,915	0.217	0.412	0.000	1.000
Sep	30,915	4.887	7.570	0.000	28.292
Inst	30,915	46.424	24.133	0.440	91.034
Dual	30,915	0.246	0.431	0.000	1.000
Board	30,915	2.262	0.180	1.792	2.773
Ind	30,915	37.066	5.241	27.270	57.140
Employee	30,915	7.579	1.290	3.970	11.264
Size	30,915	9.539	0.556	8.467	11.264
Fcf	30,915	0.046	0.073	-0.181	0.249
Roa	30,915	0.039	0.059	-0.247	0.195
Lev	30,915	0.436	0.211	0.050	0.922
Cash	30,915	0.916	1.660	0.013	11.036
MtB	30,915	0.333	0.167	0.000	0.779
Ppe	30,915	0.932	0.084	0.541	1.000
Age	30,915	1.996	0.898	0.000	3.258
Growth	30,915	0.201	0.473	-0.579	11.036

### 4.2 Propensity score matching (PSM)

#### 4.2.1 Testing the parallel trends hypothesis

**Fig 1:**
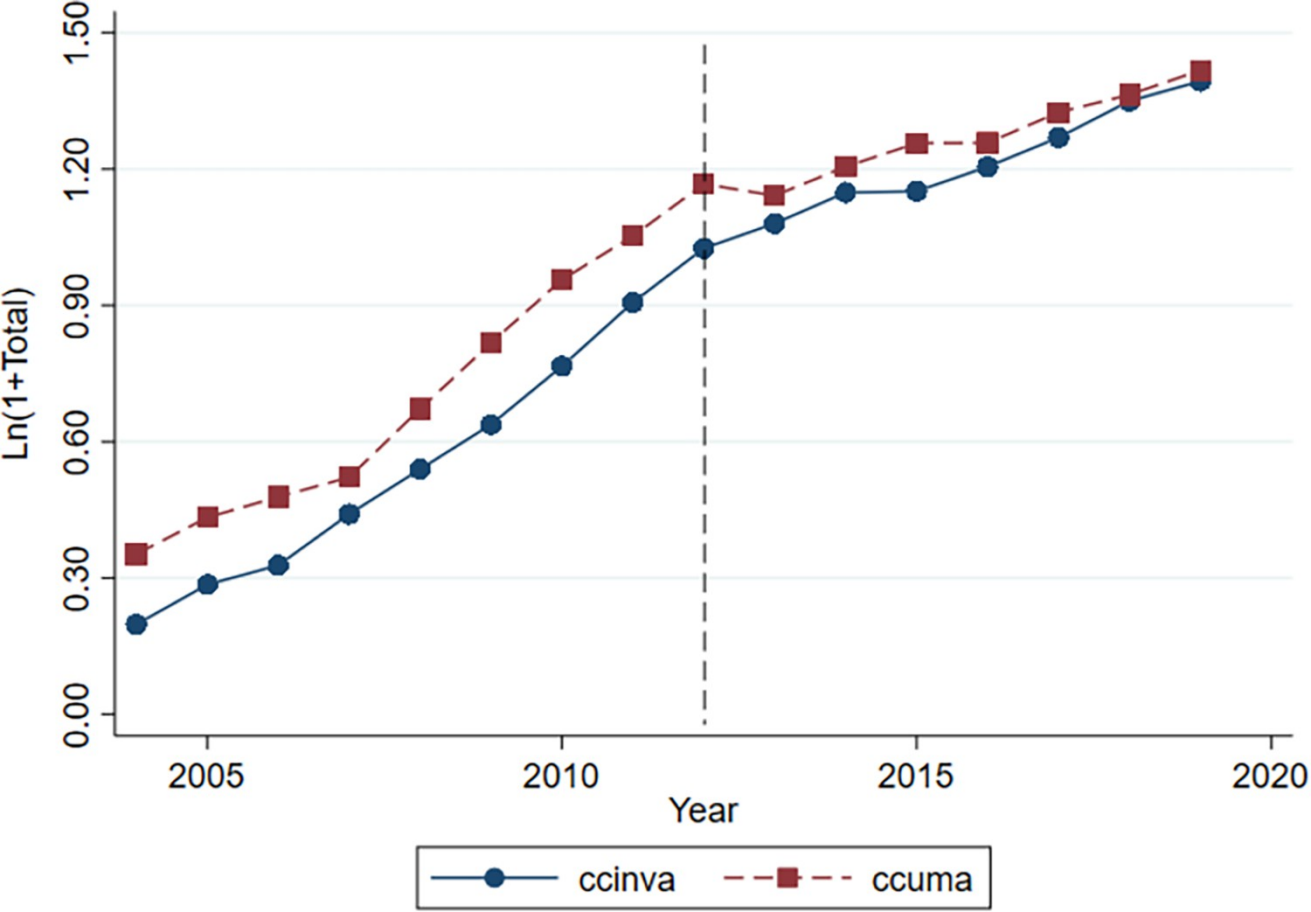
Control group.

**Fig 2:**
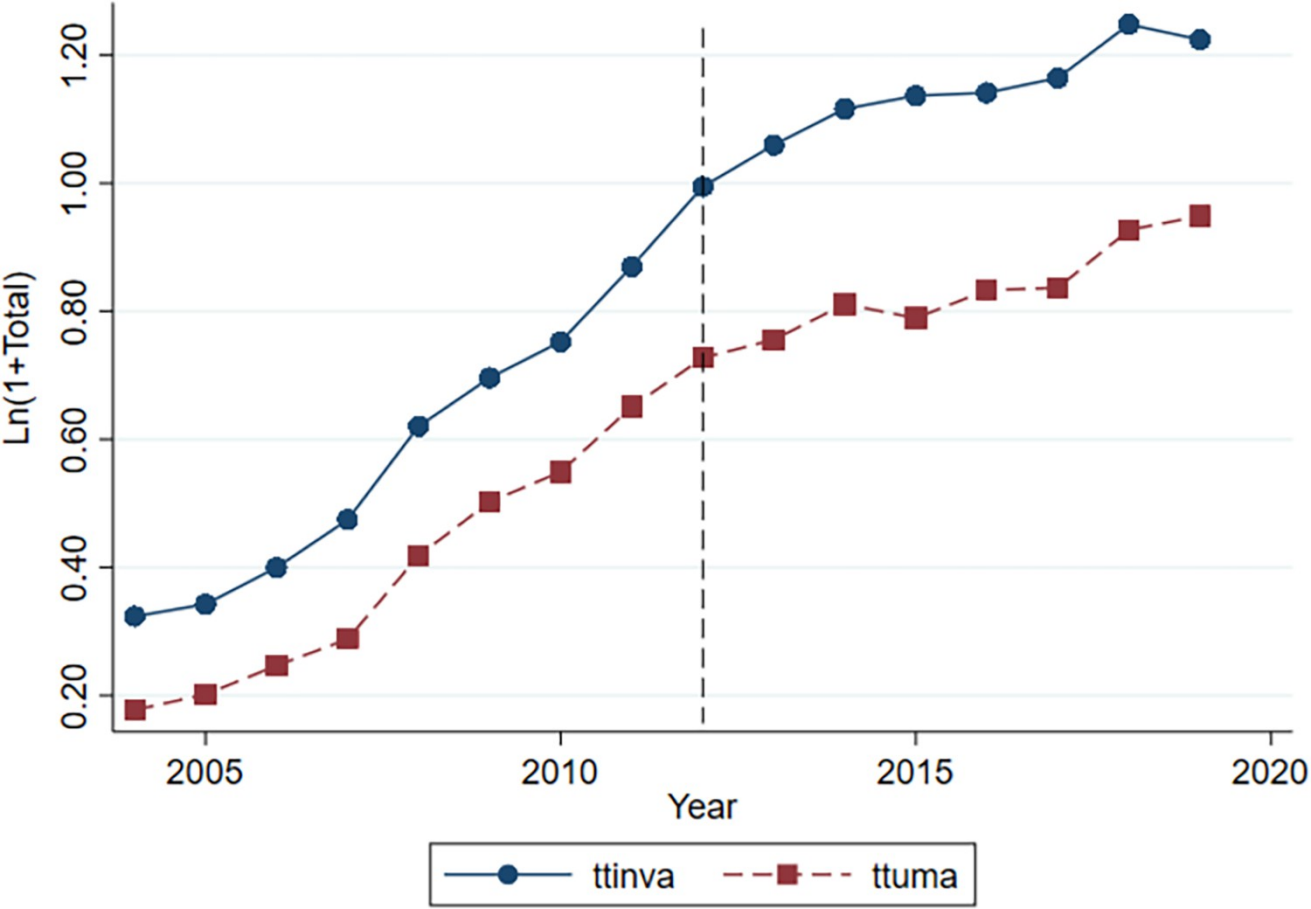
Experimental group.

Figs 1 and 2 illustrate the parallel trends of innovation output in different industries from 2004 to 2019. The horizontal and vertical axes represent the year and the innovation output of companies, respectively. The dashed line represents the Guidelines, which divides the sample into two periods: before the Guidelines (2004–2011) and after its implementation (2012–2019). The solid and dashed lines represent the number of invention patent applications and practical new-type patent applications, respectively. From Fig 1, the number of invention patents and practical new-type patents in non-restricted industries remained consistent over time before and after the Guidelines. Fig 2 shows that the average growth rate of both types of patents for green credit-restricted industries diverged significantly after the Guidelines. Thus, this finding satisfies the parallel trends assumption.

#### 4.2.2 PSM matching procedure

The DID model is an effective method for evaluating the macroeconomic policies. Based on the PSM-DID model, This study employs the nearest neighbor matching method to estimate propensity scores with a Logit model, as shown in Table 3. The covariates were matched between the experimental Group (green credit-restricted industries) and the control group (non-restricted industries). The reliability of PSM lies in whether there are significant differences between the experimental group and the control group after matching. If significant differences exist, it indicates poor matching effectiveness or inappropriate PSM matching methods, and the matching estimate is invalid. Therefore, the paper conducts a balance test for each covariate. In Table 4, the matching results show that the absolute values of standardized bias for all covariates were below 10%, and t-values were not significant. This suggests that the matching effect was good, and the estimation was valid. Thus, the matching variables and method adopted in this study are reasonable and effective, satisfying the conditions for the DID method.

**Table 3:** PSM matching results.

	Unmatched Sample	Matched Sample	Total
Control Group	13	24190	24203
Experimental Group	0	6712	6712
Total	13	30902	30915

**Table 4:** PSM balance test results.

Variable	Sample Matched	Mean		Standardized Bias %	Bias Reduction (%)	t-Value	P-Value
		Experimental Group	Control Group				
Sep	U	5.5375	4.7638	7.4	56.5	5.43	0.000
	M	5.5381	5.084	3.2		1.82	0.068
Inst	U	53.273	44.513	37.8	95.2	26.74	0.000
	M	53.286	52.891	1.8		1.11	0.269
Dual	U	0.17408	0.2724	-30.1	98.3	-20.61	0.000
	M	0.17414	0.14869	0.5		0.34	0.735
Board	U	2.3045	2.2515	26.5	90.4	19.46	0.000
	M	2.3047	2.3042	-2.6		-1.44	0.151
Ind	U	36.991	37.133	-5.9	57.6	-4.29	0.000
	M	36.99	36.691	2.5		1.48	0.138
Employee	U	8.2455	7.5377	13.8	82.7	10.76	0.000
	M	8.2461	7.6957	2.4		1.27	0.204
Size	U	9.9405	9.4592	63.8	99.8	49.72	0.000
	M	9.9413	9.8253	0.2		0.08	0.935
Fcf	U	0.04111	0.04772	-8.2	84.1	-6.20	0.000
	M	0.04113	0.04051	1.3		0.74	0.459
Roa	U	0.01496	0.4123	-20.3	82.7	-14.15	0.000
	M	0.03006	0.02776	3.5		2.03	0.042
Lev	U	0.56009	0.4042	72.3	98.5	52.21	0.000
	M	0.54095	0.5476	1.1		0.60	0.547
Mtb	U	0.30021	0.33934	-17.9	91.6	-12.72	0.000
	M	0.30048	0.30758	1.5		0.89	0.371
Ppe	U	0.9502	0.92749	28.8	97.6	19.63	0.000
	M	0.9502	0.94966	0.7		0.45	0.649
Age	U	2.1117	1.9228	39.3	92.1	27.44	0.000
	M	2.1175	2.2322	3.1		1.90	0.057
Growth	U	0.41101	0.19665	4.0	95.1	3.07	0.002
	M	0.41109	0.21765	-0.2		-0.10	0.916
Cash	U	0.59653	1.0287	-35.4	87.2	-22.80	0.000
	M	0.59672	0.57744	-4.5		-3.47	0.001

### 4.3 Benchmark regression results

Based on the established DID model, the study obtains the regression results between the green credit policy and innovation output of enterprises. Table 5 reports the benchmark regression results after introducing time and firm fixed effects. Columns (1) to (3) present the regression results for total patent applications, invention patent applications, and practical new-type patent applications, respectively.

**Table 5:** Benchmark regression results of the Guidelines on innovation performance.

	(1)	(2)	(3)
	LnTotal	LnInva	LnUma
Post	0.364***	0.341***	0.278***
	(0.026)	(0.019)	(0.022)
Treat	-0.488***	-0.354***	-0.373***
	(0.029)	(0.027)	(0.031)
Post×Treat	0.191***	-0.010	0.245***
	(0.041)	(0.036)	(0.039)
Sep	0.011***	0.009***	0.007***
	(0.001)	(0.001)	(0.001)
Inst	-0.008***	-0.007***	-0.006***
	(0.000)	(0.000)	(0.000)
Dual	0.109***	0.090***	0.057***
	(0.020)	(0.018)	(0.021)
Board	-0.121*	0.033	-0.207***
	(0.066)	(0.054)	(0.058)
Ind	0.003*	0.005***	0.003
	(0.002)	(0.002)	(0.002)
Employee	0.476***	0.324***	0.431***
	(0.010)	(0.008)	(0.009)
Size	0.595***	0.694***	0.421***
	(0.030)	(0.021)	(0.025)
Fcf	-1.536***	-1.056***	-1.531***
	(0.128)	(0.091)	(0.135)
Roa	1.290***	0.899***	0.947***
	(0.141)	(0.135)	(0.177)
Lev	-1.451***	-1.309***	-0.891***
	(0.067)	(0.056)	(0.063)
Mtb	-1.556***	-1.474***	-1.058***
	(0.063)	(0.054)	(0.053)
Age	-0.354***	-0.250***	-0.296***
	(0.011)	(0.010)	(0.011)
Growth	-0.023	-0.004	-0.016
	(0.020)	(0.016)	(0.017)
Cash	-0.042***	-0.019***	-0.047***
	(0.006)	(0.004)	(0.005)
_cons	-5.038***	-6.239***	-3.889***
	(0.274)	(0.209)	(0.216)
Year	YES	YES	YES
Id	YES	YES	YES
N	30,902	30,902	30,902
R^2^	0.240	0.229	0.198

Note: * p<0.05

** p<0.01

*** p<0.001

In Column (1), the coefficient of the interaction term Post×Treat is 0.191, which is significant at the 1% level. This indicates that the Guidelines significantly increase the total patent applications in green credit-restricted industries, highlighting the promotion effect on these industries’ innovation performance. The coefficient of Post is 0.364, which is also significant at the 1% level, suggesting that the Guidelines significantly improve the innovation performance of non-restricted industries. Meanwhile, in Column (3), the interaction term and post coefficient are both significantly positive, indicating that the Guidelines significantly increase the output of green credit-restricted industry innovation. In Columns (1) and (3), the post coefficient is greater than the interaction term Post×Treat, which indicates that for non-restricted industries, the impact of the Guidelines on enterprise innovation performance is more pronounced in this group.However, in Column (2), the coefficient of the interaction term Post×Treat is not significant, while the coefficient of Post is significant at the 1% level. This suggests that the policy effect of the Guidelines on innovation quality is heterogeneous across different industries. Specifically, the Guidelines have a significant positive impact on the innovation quality of non-restricted industries, but do not have a significant promotion effect on the green credit-restricted industries.

Based on the analysis, the study can confirm Hypothesis 1. The implementation of the Guidelines results in a significant improvement in the innovation performance of both green credit-restricted and non-restricted industries. In particular, it has a strong positive impact on the innovation output of non-restricted industries. However, its effect is limited on the number of invention patents for green credit-restricted industries. Hence, the Guidelines plays a more significant role in promoting the quantity of innovation in these industries.

### 4.4 Robustness checks

As there are various matching methods for the DID model, including nearest neighbor matching and caliper matching, this paper reports regression results after changing the matching method of the control variables from nearest neighbor matching to caliper matching. The fixed effects of the overall sample for the experimental group and control group are reported in Table 6, and the impact of green credit policy on innovation performance in different industries is compared by the regional fixed effects before and after the introduction of the Guidelines. The interaction term Post×Treat is only insignificant for invention patents, indicating that the Guidelines have a significant incentive effect on the quantity of green credit-restricted industries. The research conclusion is robust and basically consistent with Table 5.

**Table 6:** Results of robustness checks.

	LnTotal		LnInva		LnUma	
	(1)	(2)	(3)	(4)	(5)	(6)
Post×Treat	0.119***	0.117***	-0.036	-0.037	0.214***	0.212***
	(0.034)	(0.033)	(0.035)	(0.035)	(0.038)	(0.038)
Sep	-0.003*	-0.003**	-0.002	-0.003	-0.003	-0.003
	(0.002)	(0.002)	(0.002)	(0.002)	(0.002)	(0.002)
Inst	-0.000	-0.000	-0.000	-0.000	-0.001	-0.001*
	(0.001)	(0.001)	(0.001)	(0.001)	(0.001)	(0.001)
Dual	0.060***	0.057**	0.081***	0.074***	0.025	0.025
	(0.023)	(0.023)	(0.024)	(0.024)	(0.026)	(0.026)
Board	0.032	0.034	0.129	0.130	-0.028	-0.025
	(0.078)	(0.078)	(0.081)	(0.081)	(0.089)	(0.089)
Ind	-0.001	-0.001	0.003	0.003	-0.004*	-0.004
	(0.002)	(0.002)	(0.002)	(0.002)	(0.002)	(0.002)
Employee	0.180***	0.179***	0.134***	0.135***	0.203***	0.198***
	(0.017)	(0.017)	(0.018)	(0.018)	(0.019)	(0.020)
Size	0.795***	0.826***	0.876***	0.902***	0.724***	0.757***
	(0.048)	(0.049)	(0.051)	(0.051)	(0.055)	(0.056)
Fcf	0.042	0.070	0.045	0.061	-0.042	-0.008
	(0.106)	(0.106)	(0.111)	(0.111)	(0.121)	(0.121)
Roa	0.063	0.089	-0.041	-0.046	0.021	0.066
	(0.092)	(0.092)	(0.096)	(0.097)	(0.105)	(0.105)
Lev	0.025***	0.024**	0.024**	0.025***	0.021**	0.018*
	(0.009)	(0.009)	(0.010)	(0.010)	(0.010)	(0.011)
Mtb	0.038	0.031	0.060	0.057	-0.026	-0.038
	(0.062)	(0.062)	(0.065)	(0.064)	(0.070)	(0.070)
Age	0.038	0.028	0.022	0.012	0.011	0.003
	(0.025)	(0.025)	(0.026)	(0.026)	(0.028)	(0.028)
Growth	0.001	0.001	0.002*	0.002*	0.001	0.001
	(0.001)	(0.001)	(0.001)	(0.001)	(0.001)	(0.001)
Cash	0.001	0.001	0.005	0.005	0.000	0.000
	(0.003)	(0.003)	(0.003)	(0.003)	(0.004)	(0.004)
_cons	-5.971***	-6.235***	-7.588***	-7.834***	-5.867***	-6.113***
	(0.442)	(0.445)	(0.463)	(0.466)	(0.504)	(0.508)
Year	YES	YES	YES	YES	YES	YES
Id	YES	YES	YES	YES	YES	YES
Area	--	YES	--	YES	--	YES
N	30,902	30,902	30,902	30,902	30,902	30,902	30,902
30,902
R^2^	0.787	0.789	0.772	0.775	0.768	0.770

Note: * p<0.05

** p<0.01

*** p<0.001

## 5. Moderation analysis

The main effect in the study shows that the Guidelines has a significant promoting effect on enterprise innovation. Based on this, this paper draws on Aiken’s interaction effects [39] to further explore the regulatory mechanism between the two parts. By constructing the interaction terms of the Guidelines, green credit-restricted industries and the regulatory variable, the paper incorporates them into the main effect model constructed earlier to judge whether the estimated coefficient is a significant way to identify if the regulatory variable affects the main effect, that is, to explore the regulatory effect.

### 5.1 Risk-taking level

The risk-taking level of enterprises is a crucial factor in determining their financing opportunities and project selection process. Enterprises with high risk-taking levels are more likely to attract investment and other resources due to their ability to withstand risks and compensate for losses. By increasing trust between shareholders and managers, decision-makers are more inclined to seek risk. Based on the risk-taking level of each company, this paper constructs an interaction term between the risk-taking level and green credit-restricted industries before and after the Guidelines.

Table 7 reports the regulatory mechanism of the risk-taking level in the enterprise innovation performance after the Guidelines. It can be seen that the Risk×Post coefficient is significantly negative at the 1% significance level, and the Risk×Post×Treat coefficient is significantly positive at the 1% significance level. From the estimation results, the risk-taking level enhances the enterprise innovation performance by the Guidelines, but the effect varies between green credit-restricted industries and non-restricted industries. Compared to non-restricted industries, the regulatory effect is more pronounced in green credit-restricted industries. Secondly, the coefficients of the Risk×Post×Treat interaction term are all significant at the 1% level, indicating that the risk-taking level of enterprises positively moderates the impact of the Guidelines on innovation output in green credit-restricted industries. Specifically, the higher the risk-taking level of an enterprise, the more significant the incentive effect of the Guidelines on innovation upgrading. This suggests that decision-makers may be more willing to invest in technology innovation to achieve transformation and upgrading when they have higher risk-taking abilities. In Column (2) of Table 7, the coefficient of the interaction term is 0.75 and significant at the 1% level, suggesting that enterprises with higher risk-taking abilities still exhibit a willingness to increase their innovation output, even when there may be longer development cycles and greater uncertainty associated with R&D investment for invention patents in green credit-restricted industries. These findings confirm Hypothesis 2.

**Table 7:** Moderating effect of risk-taking level on innovation performance by the Guidelines.

	(1)	(2)	(3)
	LnTotal	LnInva	LnUma
Risk×Post×Treat	1.415***	0.750***	1.296***
	(0.395)	(0.273)	(0.362)
Risk×Post	-0.947***	-0.644***	-0.827***
	(0.307)	(0.212)	(0.282)
Risk×Treat	0.521*	0.201	0.505**
	(0.271)	(0.179)	(0.248)
Post×Teat	0.098*	-0.058	0.166***
	(0.057)	(0.047)	(0.053)
Risk	-0.295	-0.193	-0.264
	(0.211)	(0.139)	(0.193)
_cons	-3.694***	-4.191***	-2.790***
	(0.571)	(0.477)	(0.528)
Other variables	YES	YES	YES
N	30,902	30,902	30,902
R^2^	0.287	0.276	0.212

### 5.2 Environmental regulation

The research finds that as consumers’ pursuit gradually shifts towards green and healthy directions, the explicit cost-effectiveness declines significantly in direct environmental governance. Under environmental regulations, companies are more inclined to choose technological innovation as a way to respond actively, thereby promoting innovation output. Therefore, this paper further analyzes the regulatory mechanism of environmental regulations on the Guidelines and enterprise innovation performance. Specifically, it constructs an interaction term between environmental regulations and green credit-restricted industries before and after the Guidelines.

Table 8 reports the regulatory mechanism of environmental regulations on the enterprise innovation performance by the Guidelines. The ER×Post coefficient and the ER×Post×Treat coefficient are both significantly positive, but their coefficient differences show differences in the role of the Guidelines between green credit-restricted industries and non-restricted industries. The positive regulatory effect of environmental regulations is more pronounced in non-restricted industries. However, for green credit-restricted industries, in Columns (1) and (3), the ER×Post×Treat interaction term coefficients are 0.05 and 0.099 respectively, and are significant at the 10% and 5% levels, indicating that environmental regulations can further enhance the positive effect of the Guidelines on innovation output in green credit-restricted enterprises. Hypothesis 3 is validated.

**Table 8:** Moderating effect of environmental regulation on innovation performance.

	(1)	(2)	(3)
	LnTotal	LnInva	LnUma
ER×Post×Treat	0.050*	0.011	0.099**
	(0.074)	(0.070)	(0.081)
ER×Post	135.509***	85.476***	120.509***
	(10.947)	(8.980)	(10.034)
ER×Treat	-0.011	0.000	0.011**
	(0.046)	(0.046)	(0.048)
Post×Treat	0.128**	-0.025	0.220***
	(0.057)	(0.054)	(0.063)
ER	39.617***	24.112***	39.341***
	(5.922)	(4.764)	(5.463)
_cons	-3.480***	-4.064***	-2.647***
	(0.577)	(0.481)	(0.535)
Other variables	YES	YES	YES
N	30,902	30,902	30,902
R^2^	0.288	0.277	0.212

## 6. Heterogeneity analysis

### 6.1 Property rights heterogeneity

This paper analyzes how heterogeneity in property rights might affect the effectiveness of green credit policies in China. State-owned enterprises are generally considered more credible in financing opportunities from banks and other financial institutions, which could result in different policy effects between state-owned and non-state-owned enterprises under this Guidelines. Therefore, this study divides the sample into state-owned and non-state-owned enterprises based on property rights and compares their innovation levels before and after the Guidelines. Table 9 reveals that, compared to non-state-owned enterprises, state-owned enterprises have significant differences in their innovative output restricted by green credit policies before and after the Guidelines, resulting in more significant improvements in their innovation performance. However, there remains a lack of incentive for innovation quality. These findings indicate that the Guidelines has more significant effects on state-owned enterprises, supporting the validation of Hypothesis 4.

**Table 9:** Results of the property rights heterogeneity analysis.

	Government-owned enterprise			Private enterprise		
	(1)	(2)	(3)	(4)	(5)	(6)
	LnTotal	LnInva	LnUma	LnTotal	LnInva	LnUma
Post×Treat	0.120* (0.072)	-0.065 (0.073)	0.224*** (0.078)	0.104 (0.087)	0.016 (0.079)	0.165* (0.099)
_cons	-7.997*** (1.280)	-9.596*** (1.268)	-7.684*** (1.563)	-7.586*** (0.935)	-8.722*** (0.987)	-7.532*** (1.042)
Year	YES	YES	YES	YES	YES	YES
Area	YES	YES	YES	YES	YES	YES
N	13,272	13,272	13,272	17,630	17,630	17,630
R^2^	0.288	0.264	0.256	0.225	0.199	0.196

### 6.2 Size heterogeneity

In this paper, the median of the sample size is used as the threshold to distinguish small-scale enterprises from large-scale ones. The study examines whether differences in enterprise size affect the effectiveness of the Guidelines. Regression results in Table 10 indicate that, after the Guidelines, innovation performance notably improves for large-scale enterprises compared to their smaller counterparts, especially in terms of patent output. This supports Hypothesis 5.

**Table 10:** Results of size heterogeneity analysis.

	Large-scale enterprise			Small-scale enterprise			
	(1)	(2)	(3)	(4)	(5)	(6)	
	LnTotal	LnInva	LnUma	LnTotal	LnInva		LnUma
Post×treat	0.200*** (0.077)	0.001 (0.068)	0.242*** (0.072)	-0.016 (0.087)	-0.045 (0.070)		0.045 (0.082)
_cons	-5.468*** (1.201)	-5.636*** (1.057)	-4.289*** (1.121)	-6.447*** (0.840)	-5.540*** (0.706)		-5.038*** (0.774)
Year	YES	YES	YES	YES	YES		YES
Area	YES	YES	YES	YES	YES		YES
N	15,451	15,451	15,451	15,451	15,451		15,451
R^2^	0.272	0.231	0.261	0.172	0.152		0.131

## 7. Conclusion and suggestions

The study enriches the relevant research on the effect of green credit policy on stimulating enterprise innovation, which finds that after the Guidelines, the overall innovation performance of enterprises has been improved, and it is more pronounced in non-restricted industries. However, this promotion effect is only manifested in the numbers of innovation output, and the effect on innovation quality is not significant. Furthermore, since innovation needs high input and long cycles, the ability to bear unknown risks is one of the factors that affect the enterprise innovation. Better risk-taking abilities can enhance the incentive effect of green credit policy on enterprise innovation output. From an external perspective, although the stronger in environmental regulatory leads to an increase in compliance cost expenditures for companies, the innovation compensation effect will bring about a rise in capital returns in the long run. Secondly, through heterogeneity analysis, the paper finds that differences in property rights and enterprise size also effect the innovation performances of differen enterprises by the Guidelines, among which state-owned and large-scale green credit-restricted enterprises are more sensitive to this policy, and the promotion is more significant.

This study holds some the policy implications that green credit policy can better promote enterprise innovation performance, which is a win-win policy that advances the coordination of environment and economy, but its policy effect needs to be fully realized with a better matching institutional system. According to the mechanism analysis, better the risk-taking capacity and environmental regulations are both ways to further enhance the Guidelines effect on innovation performance. The green credit policy plays an important role in the realization of China’s 3060 dual carbon goals, and contributes to the development strategy of an innovative and environment friendly country. Therefore, the following recommendations are proposed:

1. In order to seek upgrading measures to meet social needs, enterprises play the leading role in innovation with social responsibilities. Firstly, they should public the environmental Information actively for the business goal of environmental and economic benefits. Secondly, avoiding excessive reliance on indirect borrowing from banks, enterprises would seek for more financing channels as venture capital funds and government support with strong risk management to reduce the uncertainty of innovative projects. Moreover, they also need full use of funds invested in research actively for innovative transformation.
2. The government focuses on the effectiveness of credit policies and improves a more sound green credit system. Firstly, based on regional differences in development and capabilities, differentiated green credit systems should be established, tailored to local conditions and enterprises. Thus, differential development routes are created to match financial supply. Secondly, the green credit market would be expanded to stimulate the enterprise innovation enthusiasm and connect banks with other green finance markets as carbon finance, thus providing more financial support for green innovative enterprises with an information sharing platform. Furthermore, the research shows that mandatory environmental regulations can further enhance the effect of credit policies on innovative transformation. Therefore, various environmental regulatory measures should be combined to an incentive guidance mechanism, which will provide more informations under a reasonable evaluation system and increase the cost of rent-seeking behaviors with mandatory measures.
3. Based on comprehensive and accurate environmental and risk assessment, financial institutions should establish differentiated credit management systems through digital information platforms, forming a dynamic adjustment system of credit resources. This system could solve the information asymmetry and lift the constraint on restricted enterprises with different treatments, and aim to provide more suitable and effective financial support for enterprises.

## Supporting information

S1 Data Regression data. (XLSX)
